# Mast Cells Mediate Acute Inflammatory Responses After Glenoid Labral Tears and Can Be Inhibited With Cromolyn in a Rat Model

**DOI:** 10.1177/03635465241278671

**Published:** 2024-10-06

**Authors:** Cynthia M. Co, Bhavya Vaish, Le Q. Hoang, Tam Nguyen, Joseph Borrelli, Peter J. Millett, Liping Tang

**Affiliations:** * Department of Bioengineering, University of Texas at Arlington, Arlington, Texas, USA; † Department of Orthopedic Surgery, The Steadman Clinic, Vail, Colorado, USA; # These authors contributed equally to this article; Investigation performed at the University of Texas at Arlington, Arlington, Texas, USA

**Keywords:** cromolyn, glenoid, labrum, mast cells, synovium

## Abstract

**Background:**

Injuries to the glenoid labrum have been recognized as a source of joint pain and discomfort, which may be associated with the inflammatory responses that lead to the deterioration of labral tissue. However, it is unclear whether the torn labrum prompts mast cell (MC) activation, resulting in synovial inflammatory responses that lead to labral tissue degeneration.

**Purpose:**

To determine the potential influence of activated MC on synovial inflammatory responses and subsequent labral tissue degeneration and shoulder function deterioration in a rat model by monitoring MC behavior and sequential inflammatory responses within the synovial tissue and labral tissue after injury, suture repair, and MC stabilizer administration.

**Study Design:**

Controlled laboratory study.

**Methods:**

Anteroinferior glenoid labral tears were generated in the right shoulder of rats (n = 20) and repaired using a tunneled suture technique. Synovial tissue inflammatory responses were modulated in some rats with intraperitoneal administration of an MC stabilizer—cromolyn (n = 10). At weeks 1 and 3, MC activation, synovial inflammatory responses, and labral degeneration were histologically evaluated. Simultaneously, gait analysis was performed before and after surgical repair to assess the worsening of the shoulder function after the injury and treatment.

**Results:**

Resident MC degranulation after labral injury (50.48% ± 8.23% activated at week 1) contributed to the initiation of synovial tissue inflammatory cell recruitment, inflammatory product release, matrix metalloproteinase-13, and subsequent labral tissue extracellular matrix degeneration. The administration of cromolyn, an MC stabilizer, was found to significantly diminish injury-mediated inflammatory responses (inflammatory cell infiltration and subsequent proinflammatory product secretion) and improve shoulder functional recovery.

**Conclusion:**

MC activation is responsible for labral tear–associated synovial inflammation and labral degeneration. The administration of cromolyn can significantly diminish the cascade of inflammatory reactions after labral injury.

**Clinical Relevance:**

Our findings support the concept that MC stabilizers may be used as a complementary therapeutic option in the treatment and repair of labral tears.

The glenoid labrum is a fibrocartilage ring surrounding the glenoid cavity that participates in shoulder joint stability. Glenoid labral tears are often encountered in overhead athletes and can occur over multiple regions around the glenoid. Traumatic anterior shoulder dislocation often results in the detachment of the anteroinferior labrum.^48^ Superior labrum anterior-posterior (SLAP) tears are common injuries related to joint overuse and trauma and are associated with poor clinical outcomes in young and active individuals.^24,36^ Batter shoulder is a condition affecting baseball players in which the posterior labrum is injured from throwing or diving with a fully extended arm.^26^ Injuries to the glenoid labrum can lead to chronic pain, persistent shoulder instability, and low rates of returning to the previous level of play.^26^ Unfortunately, failure of labrum healing after arthroscopic repair has been associated with deterioration of the labral tissue and joint instability, a major risk factor for the development of osteoarthritis.^5,23,43^ However, it is not clear how labral tears contribute to labral degeneration.^11,38,39^ It is generally believed that inflammatory processes in the joint, such as those associated with osteoarthritis or rheumatoid arthritis, participate in the development and progression of labral degeneration after injury.^8,35,40^

The labrum is in direct contact with the synovial membrane lining the inner surface of the shoulder joint. While the synovial fluid produced by the synovial tissue is responsible for lubricating and nourishing the articulating structures within a diarthrodial joint, it may also serve as an avenue for inflammatory cytokines and subsequent tissue destruction.^4^ Synovial inflammation has been associated with the degeneration of other tissues, such as the rotator cuff, cartilage, meniscus, and acetabular labrum.^4,9,10,29,31^ For example, interleukin-1 beta (IL-1β) and tumor necrosis factor-alpha (TNF-α) have been found to be the main proinflammatory cytokines associated with synovial inflammation that accelerates the catabolism of cartilage in knee osteoarthritis.^46,47^ In synovial tissue of patients with rheumatoid arthritis, proinflammatory enzyme matrix metalloproteinase 13 (MMP-13) has been detected in macrophage- and fibroblast-like cells, in vascular endothelial cells, and the membrane lining.^33^ More importantly, high messenger ribonucleic acid expression of TNF-α, IL-1β, IL-6, and cyclooxygenase-2 have been found in synovial tissues of patients with degenerative acetabular labral tears.^29^ Moreover, high quantitative polymerase chain reaction levels of TNF-α, IL-1β, ADAMTS4, MMP-1, and MMP-3 in synovial membranes of patients undergoing hip arthroscopy for labral tears have also been found and are believed to be associated with tissue degeneration.^19^ Despite these observations, little is known about whether synovial inflammation might contribute to glenoid labral degeneration after a labral tear.

Mast cells (MCs) are immune cells found near blood vessels and broadly distributed in connective tissue and mucosa, such as synovial tissue. While MCs are traditionally associated with allergic responses, they also play versatile roles in the immune system, including their involvement in various inflammatory processes beyond allergic reactions. For example, MCs found within synovium have been implicated in the pathogenesis of inflammatory joint diseases, such as rheumatoid arthritis and osteoarthritis.^7^ Synovial MC activation or degranulation has been shown to trigger neutrophil infiltration into sites of local inflammation and subsequent destruction of several tissues—including skeletal muscle and articular cartilage.^51,53^ MC granule products—including histamine, proteases, cytokines, and chemokines—are well established to promote immune cell infiltration, inflammatory responses, and tissue injury.^16^

While the presence of MCs in synovial tissue has been documented in the joints,^17,22^ little is known about whether MC activation may contribute to synovial inflammation in the glenohumeral joint after labral tear/injury. We hypothesized that synovial MC activation-mediated inflammatory responses would be responsible for labral degeneration and shoulder function deterioration after glenoid labral injury. To test this hypothesis, we used a labral tear rat model, which has been shown to simulate glenoid labral pathology in humans.^11,38,39^ The role of MC activation on synovial inflammation and labral degradation was examined by modulating MC activation through the administration of cromolyn, an MC stabilizer, which has been shown to effectively reduce MC degranulation and thus reduce MC activation-associated inflammatory responses.^20^

## Methods

### Study Design

In this proof-of-concept study, we used 40 adult Sprague-Dawley rats (M/F, 300-500 g; Charles River Laboratories). The animals were randomly divided into 4 groups, with 5 rats in each group and 2 endpoints (1 and 3 weeks): (1) healthy; (2) sham surgery; (3) injured; and (4) injured + cromolyn. In addition, to establish positive controls, healthy shoulders (3 rats each) were given compound 48/80.

All animals were treated according to the standard guidelines approved by the Institutional Animal Care and Use Committee at the University of Texas at Arlington in accordance with the Animal Welfare Act and the Guide for Care and Use of Laboratory Animals.

### Surgical Procedure

**Figure 1.**
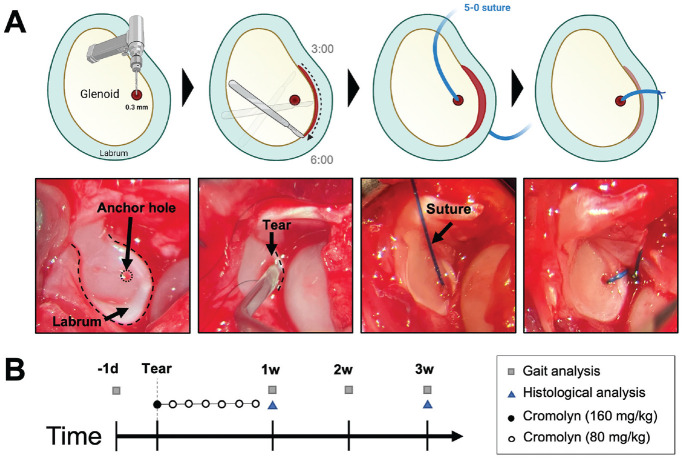
Schematic of surgical procedure/study design. (A) Glenoid labral tears were generated in rat shoulders—3 and 6 o’clock positions in reference to the right shoulder—and repaired using suture anchors. (B) Functional evaluation was performed on all groups before and weekly after surgery. Rats were administered 9 dosages of cromolyn starting the day before surgery, the day of surgery, and daily for 1 week after surgery. The control group received saline injections. Rats were sacrificed for histological analysis at 1 and 3 weeks.

Glenoid labral tears were created in rats using previously described methods, with a modification to use a suture via bone tunnel for tear repair (Figure 1A).^11,38,39^ Briefly, rats were anesthetized using isoflurane inhalation, an intramuscular dose of cefazolin and subcutaneous dose of Buprenorphine SR was then administered. An incision of 3 to 4 cm was made longitudinally along the posterior aspect of the right shoulder after shaving and betadine sterilization. The deltoid was incised in parallel to its fibers posteriorly to expose the posterior rotator cuff. The rotator cuff and capsule were then incised longitudinally to expose the glenohumeral joint. The shoulder was dislocated anteriorly and inferiorly until the humeral head was completely dislocated on the glenoid. The dislocation created an instability model, simulating the nonrepaired shoulder complex laxity encountered clinically. Traction was applied to the forelimb to expose the glenoid labrum. Using a 0.3-mm diameter tungsten carbide drill bit, a tunnel site was produced to prepare the glenoid for labral repair via suture. A hole was drilled through the glenoid cavity directly under the labrum so that the drill hole would be perpendicular to the labral tear. A 5-0 nonabsorbable suture (Prolene Polypropylene; Ethicon) was fed through the drill hole and joint capsule and pulled above the surface of the labrum. Next, an ophthalmic blade was used to create a small laceration in the anteroinferior labrum (4- and 5-o’clock positions in reference to the right shoulder) at the level of the glenoid rim (1 mm length). Then, the labrum was mechanically torn using a surgical probe for complete detachment of the labrum from bone of identical sized tears (~2 mm length), which included the entire anterior glenoid labrum (3 o'clock position) continuing inferiorly to a point just past the 6 o'clock position on the glenoid face, with the 12 o'clock position designating the insertion of the biceps tendon into the supraglenoid tubercle. Finally, the suture was tightened with the knot resting on the capsular edge of the labrum to prevent it from articulating with the humeral head. The shoulder was relocated, the rotator cuff and subcutaneous tissue were repaired with 4-0 nonabsorbable sutures, and the skin was closed using wound clips. Once fully awake, the rats received a subcutaneous dose of meloxicam, a nonsteroidal anti-inflammatory drug (1 mg/kg), readministered once every 24 hours for 3 days. The rats were monitored every 24 hours for the first 3 days after surgery and then weekly. Buprenorphine SR, 1.2 mg/kg, was readministered after 3 days. To differentiate the effect of the labral tear from the overall impact/trauma of the surgery, sham surgeries were performed (n = 3) using the same procedure above without creating the labral tear or suture anchor. Rats were allowed free cage activities and additional enrichment.

To test the hypothesis that MCs trigger inflammatory responses leading to labral degeneration, we attenuated MC activation using pharmaceutical grade cromolyn sodium (Sigma Aldrich)—an antiasthmatic and MC stabilizer. The dosing pattern was based on several previous publications.^2,12,15^ Animals were given daily intraperitoneal injections of cromolyn (dissolved in sterile saline). The first cromolyn dose (160 mg/kg in saline) was administered 1 day before the surgery. The animals were treated with 80 mg/kg cromolyn on the day of the surgery and daily the following 7 days (Figure 1B). In another set of animals, we administered 2 mg/kg of a well-known MC activator (compound 48/80; Sigma Aldrich) locally to the healthy shoulder joint without a surgical procedure^30^ (n = 3). Controls received saline.

### Histological Analysis

The soft tissue surrounding the glenohumeral joint was removed from all euthanized animals without damaging the joint capsule, and the joint capsule was separated from its humeral insertions. Healthy shoulders with no surgical procedure or treatment were used as controls. The treatment assignments were kept undisclosed to ensure that the technicians who conducted the tissue analysis were unaware of the treatment assignments during material implantation. The tissue samples from both the healthy and injured shoulders were fixed in a buffered paraformaldehyde solution of 4% w/v, decalcified with ethylenediaminetetraacetic acid at 10% w/v for approximately 2 weeks, embedded in paraffin wax, and then manually sectioned using a rotary microtome. Toluidine blue staining and immunohistochemical (IHC) staining using the antibody against MC tryptase (sc-59587; Santa Cruz Biotechnology)—an enzyme found predominantly in MC secretory granules—were performed to confirm MC activation.^50^ Toluidine blue stains the MC granules purple-to-blue with unique recognizable cytoplasmic metachromatic granules only found in MCs.^44,45^ Stained MCs that were flat and rough with obvious granular product release were defined as activated, whereas inactivated naïve MCs of healthy joints appeared rounder and smoother.^18^ Hematoxylin and eosin Y staining was performed for morphological assessment and cell density quantification, and IHC staining was done to evaluate inflammatory responses using antibodies against CD11b (sc-6614; Santa Cruz Biotechnology), proinflammatory cytokines—MMP-13 (18165-1-AP; Thermo Fisher Scientific) and IL-1β (BS-20449R; Thermo Fisher Scientific). Diaminobenzidine, a chromogen, was used to develop the color for CD11b+, MMP-13+, and IL-1β+ staining. Toluidine blue was used to evaluate labral glycosaminoglycan (GAG) composition.^11,32^ Images were acquired using a light microscope and analyzed in and around the injured labrum using ImageJ software (National Institutes of Health).^11,32^ The location of the histological samples is the shoulder joint, with labral tissue in the middle and surrounded by synovial and bone tissue. We quantified a 1 mm^2^ region of interest in the synovial tissue directly adjacent to the labrum, and the percentage of activated MCs was calculated using the following equation: MC_#activated_/MC_#total_× 100%. The extent of inflammatory responses was evaluated based on the number of inflammatory cells (CD11b) and the intensity of inflammatory enzyme (MMP-13) and chemokine (IL-1β) staining. The intensity of GAG staining was measured to evaluate labral degeneration. No animals were excluded from the analysis (n = 5 for each analysis).

### Functional Assessment

Gait analyses were conducted before and weekly after surgery for up to 3 weeks according to previous publications (Figure 1B).^3,6,25,27,41^ To produce paw prints, rats were placed on an ink pad and confined to a 24 × 4 inch (length × width) transparent walkway underlined by white paper. There was a 2-week acclimatization period before the measurements, which were performed at the same time of day and by the same handler (L.Q.H.). A ruler was used to measure the stride length (length from ipsilateral to ipsilateral paw) and the ipsilateral to contralateral step length, where the ipsilateral limb refers to the forelimb on the same side as the operation. Because each rat produced multiple step lengths and stride lengths on its gait sheet, a single rat's “step length” was taken as the mean of all step lengths on its gait sheet, whereas a single rat's “stride length” was taken as the mean of all stride lengths on its gait sheet. The step length difference per week was defined as the current step length minus the baseline step length (step length before surgery), and the stride length difference for each week was defined as the current stride length minus the baseline stride length (stride length before surgery). This was done to account for the rat-to-rat variations. Gait sheets with paw prints were scanned in grayscale using an Epson Expression 10000XL scanner and placed in ImageJ to measure the ipsilateral and contralateral paw areas. The ipsilateral to contralateral paw area for each rat was calculated to account for the rat-to-rat variations. Those performing the gait analysis were unaware of the treatment allocation.

### Statistical Analysis

All statistical analyses were performed using GraphPad Prism Version 9.0.0 (GraphPad Software). The sample size was calculated using G*Power, with a power of 0.80 and an α value of .05 using previous results (based on MC activation) from our pilot study in which labral tears were generated in rats (n = 3) and analyzed after 1 week. Comparisons of injured synovial and labral tissues were made using 1-way analysis of variance (ANOVA) and Tukey post hoc tests and were considered statistically significant when *P*≤ .05. The normality and homoscedasticity were confirmed using the Shapiro-Wilk normality and Bartlett tests, respectively. As described earlier, a linear regression was performed to analyze the relationship between MC numbers and CD11b+ inflammatory cell infiltration.^13^ Statistical analysis for gait analysis was conducted using the appropriate 1-way ANOVA and post hoc analysis. Specifically, for a comparison of groups every week, the samples (or each group’s data) were analyzed for normality and homogeneity of variances, and 1 of the following analyses was conducted based on sample normality and variances: 1-way ANOVA with Tukey test (normal samples with homogeneous variances), 1-way ANOVA with the Games-Howell test (normal samples with nonhomogeneous variances), or 1-way ANOVA on ranks with Dunn test (not normal) The results are presented as mean and standard deviation. The study data are available upon request.

## Results

### Synovial MC Activation and Inflammatory Responses

**Figure 2.**
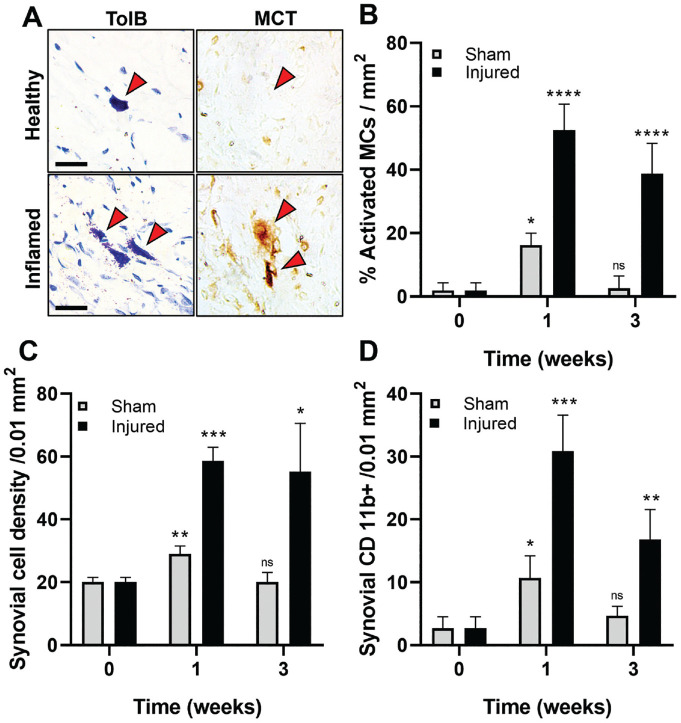
Characterization of synovial tissue inflammatory response 1 and 3 weeks after sham surgery and labrum injury. (A) Abundant activated MCs—identified with toluidine blue and MCT—were found in synovial tissue near the injured labrum but not in healthy control. Scale bar = 25 µm. (B) The percentage of activated MCs, (C) the density of synovial cells, and (D) the density of CD11b+ inflammatory cells in synovium were quantified at different time points. Data are presented as mean ± standard deviation. MCs, mast cells, MCT, mast cell tryptase; Ns, not significant. *P < .05; **P < .001; ***P < .001; ****P < .0001.

To test the hypothesis that MC activation triggers inflammatory cell infiltration, proinflammatory exudation, and subsequent labral degeneration, we first examined the MC distribution and activation status in healthy shoulder joints. While no MCs were present in healthy labral tissue, there were a few MCs in the synovium, often located near vasculature and muscle surrounding the glenohumeral joint. On the other hand, histological examination of injured shoulders revealed many activated MCs in the synovium of labral tears near the vasculature (Figure 2A). MC tryptase, an enzyme found predominantly in MC secretory granules, confirmed MC activation (Figure 2A). The number of synovial and activated MCs significantly increased and spread throughout the synovium from vasculature toward the injured labrum. MC activation reached the highest levels in injured tissue at week 1 with 50.48% ± 8.23% activated MCs, while 1.88% ± 2.40% activated MCs were found in healthy tissue. (Figure 2B). At week 3 after surgery, the percentage of activated MCs in animals with labral injury only reduced slightly compared with week 1. MC activation coincided with a significant accumulation of cells in the synovium (Figure 2C), with a 3-fold increase of synovial cellularity by week 1 after injury compared with healthy synovia (58.60 ± 4.39 vs 20 ± 1.58 cells/0.01 mm^2^). Using IHC staining, we assessed the effect of synovial MC activation on CD11b+ inflammatory cell recruitment in injured synovial tissue. The number of CD11b+ cells was significantly higher (~7×) in injured synovial tissue than in healthy controls at week 1 (Figure 2D).

Additional analyses were performed on tissue with sham surgery to determine the cause of MC activation. Sham surgery (no labral tear) alone was found to trigger mild short-term (1 week) MC activation and inflammatory responses. There was 3× (*P* < .0001) less synovial MC activation in animals with sham surgery than with labral tears (Figure 2B). By week 3, the extent of MC degranulation (2.70% ± 4.81%) in the sham surgery group was like that of healthy controls. Similar trends were also found with the synovial cell density and CD11b+ inflammatory cell infiltrations (Figure 2, C and D). These results suggest that sham surgery alone only has acute and transient effects on synovial MC activation and inflammatory responses. To directly verify the role of MC activation in synovial inflammatory responses, a control study was performed in which some healthy shoulder joints were intra-articularly administered with an MC activator, compound 48/80, or saline. The results show that a single injection of 48/80 triggered profound MC activation and inflammatory responses in the synovium at 24 hours (Appendix Figure A1, available in the online version of this article). By correlating the MC and inflammatory cell responses in the synovium, we found an excellent linear relationship between activated MC numbers and CD11b+ inflammatory cell numbers in the synovium (*R*^2^ = 0.9809). The results further confirm the pivotal role of MC activation in synovial inflammatory responses after labral tear and injury.

**Figure 3.**
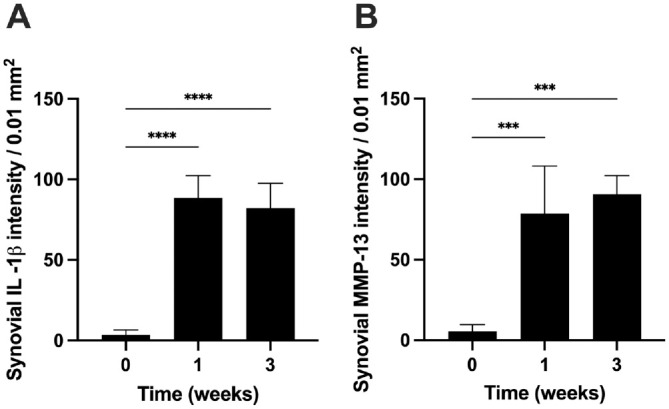
Synovial inflammatory responses were quantified 1 and 3 weeks after labral injury. (A) IL-1β intensity, and (B) MMP-13 intensity in the synovium after labral injury. Data are presented as mean ± standard deviation. n = 5. IL-1β, interleukin-1 beta; MMP-13, matrix metalloproteinase. ***P < .001; ****P < .0001.

Equally important, we found that the immigrated inflammatory cells in the synovial tissue also produced statistically higher concentrations of cytokines and tissue degradation enzymes, such as IL-1β (~8×) and MMP-13 (~7×), in the injured labrum group than those in healthy control at both weeks 1 and 3 (Figure 3).

### Labral Inflammatory Responses and Tissue Degeneration

**Figure 4.**
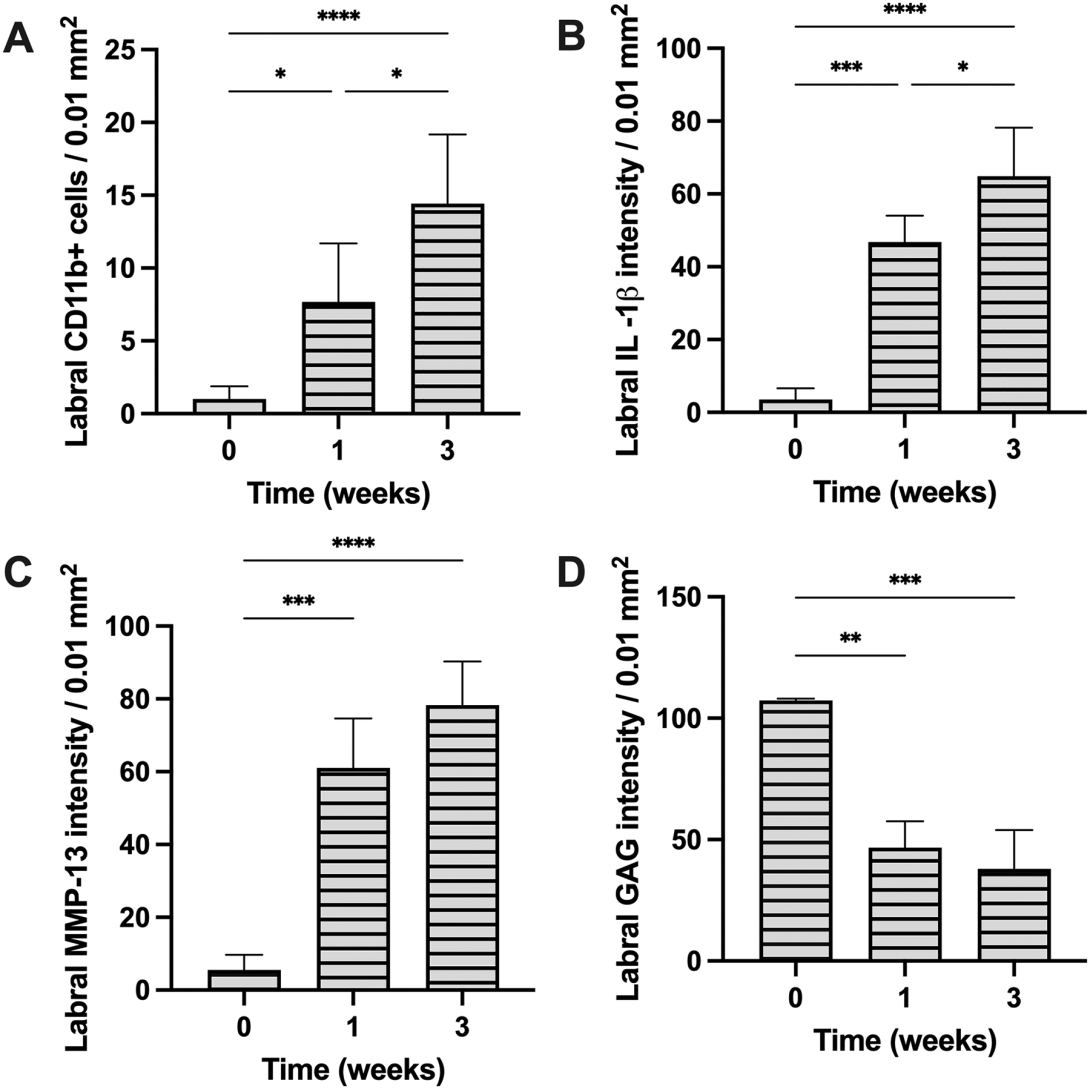
The extent of inflammation and degeneration in torn labrum tissue 1 and 3 weeks after labral injury. (A) CD11b+ cell density, (B) IL-1β intensity, (C) MMP-13 intensity, and (D) GAG intensity in the torn labrum at different time points. Data are presented as mean ± standard deviation. GAG, glycosaminoglycan; IL-1β, interleukin-1 beta; MMP-13, matrix metalloproteinase; n = 5. *P < .05; **P < .01; ***P < .001; ****P < .0001.

The increasing inflammatory responses in synovial tissue also produced an inflammatory and tissue degenerative effect on torn labral tissue. Specifically, in labral tissue after injury, we observed an increase in inflammatory cells (CD11b+ cells) and cytokine production (IL-1β and MMP-13). Labral CD11b+ inflammatory cell density increased ~5× and ~7× after 1 and 3 weeks, respectively (Figure 4A). The increase of inflammatory cells is also accompanied by increased inflammatory product release. Specifically, compared with week 0, torn labral tissues exhibited ~4× and ~12× increase in IL-1β concentrations at weeks 1 and 3, respectively (Figure 4B). Torn labra were also found to contain significantly higher MMP-13 concentration than healthy controls (week 0) (6× at week 1 and ~8× at week 3) (Figure 4C). Most importantly, our results showed a large reduction (~50%) in labral GAG composition as early as 1 week compared with healthy labral tissue (Figure 4D).

### Source and Mechanism of Injury-Mediated Labral Inflammation

**Figure 5.**
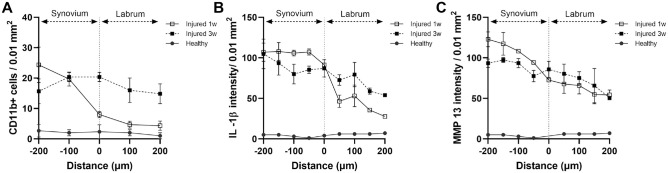
Origin of labral inflammation after labral tear injury and synovial inflammation. Spatial distribution of (A) CD11b+ inflammatory cells, (B) IL-1β, and (C) MMP-13 across the capsulolabral complex after 1 and 3 weeks. Data are presented as mean ± standard deviation. IL-1β, interleukin-1 beta; MMP-13, matrix metalloproteinase; n = 5.

Because the synovial membrane, but not the labrum, is composed of synovial MCs and a vascular network that serves as a key passage for inflammatory cells, we thus hypothesized that labral injury–mediated inflammatory responses would be governed by a 2-step process. First, synovial MC activation prompts inflammatory cell recruitment toward the torn labrum. Second, the products released by the inflammatory cells contribute to labral tissue degradation. The distribution of CD11b+ cells across both synovial and labral tissue was quantified to test this hypothesis. As expected, while healthy synovia and labra contain minimal CD11b+ cells, we found that many CD11b+ cells were present in the injured synovial tissue, and the density of CD11b+ cells reduced at the edge of and inside the torn labrum at 1 week (Figure 5A). At 3 weeks, CD11b+ cell density in the labrum continued to increase, while the synovial CD11b+ cell density decreased. This trend reversal suggests that the CD11b+ cells initially populated in the inflamed synovium likely migrated into torn labral tissue. Similarly, the distribution of inflammatory products across the tissue showed that the concentrations of IL-1β and MMP-13 were higher in the synovial tissue and moved to the labral region (Figure 5, B and C). The overall results suggest MCs play a pivotal role in the degeneration of torn labrum via a 3-step process. First, labral injury prompts the activation of synovial MC. Second, MC products trigger the immigration of inflammatory cells from synovial vasculature toward torn labrum tissue. Third, the inflammatory cells release cytokines and enzymes that lead to labral inflammation and, finally, labral tissue degradation.

### Effect of MC Stabilization on Synovial and Labral Inflammation

To investigate the role of MCs in synovial and labral inflammation and tissue degeneration, 10 animals with labral injury were treated with cromolyn (n = 5 per time point) to inhibit the degranulation of synovial MCs. Saline injections were given as controls. After 1 and 3 weeks, synovia were analyzed for MC activation and inflammatory responses, and labra were analyzed for degeneration.

**Figure 6.**
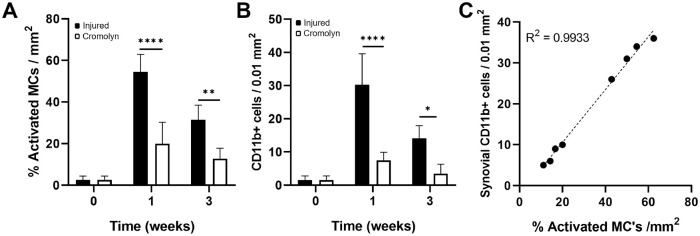
Effect of cromolyn treatment on inflamed synovial tissue. (A) Percentage of activated MCs and (B) density of CD11b+ cells. (C) Linear regression between activated MCs and synovial CD11b+ in injured synovial tissue with or without treatment. Data are presented as mean ± standard deviation. MCs, mast cells; n = 5. **P < .01; ***P < .001; and ****P < .0001.

Regarding synovial inflammatory responses, cromolyn treatment reduced synovial MC activation after labral injury by ~40% and ~30% at 1 and 3 weeks, respectively (Figure 6A). Subsequently, overall synovial cellularity stayed elevated for at least 3 weeks in nontreated tissues and was reduced with cromolyn treatment at 1 and 3 weeks. IHC staining revealed that CD11b+ inflammatory cell infiltration in the synovium was significantly reduced by 3× at both time points with MC stabilization (Figure 6B). The linear regression analysis showed a high correlation (*R*^2^ = 0.9933) between the number of activated MCs and CD11b+ inflammatory cell infiltration in the synovium (Figure 6C). These results support that MC activation is responsible for inflammatory cell recruitment.

**Figure 7.**
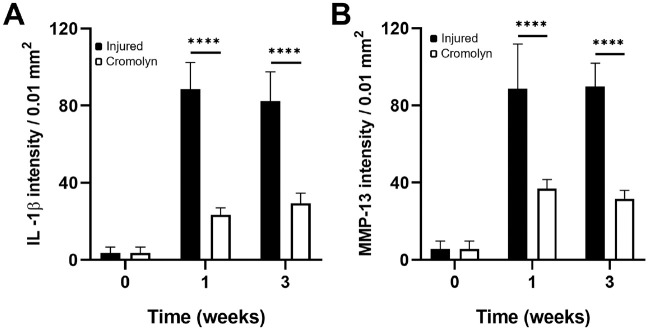
Effect of cromolyn treatment on (A) IL-1β intensity and (B) MMP-13 in inflamed synovial tissue with or without treatment. Data are presented as mean ± standard deviation. IL-1β, interleukin-1 beta; MMP-13, matrix metalloproteinase; n = 5. ****P < .0001.

MC stabilization also significantly reduced the production of inflammatory products IL-1β (injured vs cromolyn = 88.5 ± 13.8 vs 23.4 ± 3.7 at week 1; 82.4 ± 15.1 vs 29.3 ± 5.3 at week 3) and MMP-13 (injured vs cromolyn = 88.5 ± 23.3 vs 37 ± 4.6 at week 1; 89.9 ± 12.1 vs 31.7 ± 4.3 at week 3) in the synovia at both time points, respectively (Figure 7).

**Figure 8.**
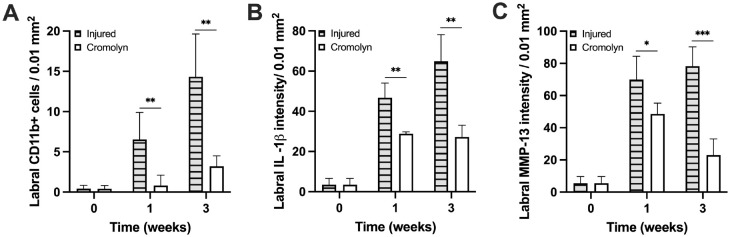
Influence of cromolyn treatment on the inflammatory responses of injured labral tissue. (A) CD11b+ cell density, (B) IL-1β intensity, and (C) MMP-13 intensity in injured labral tissue with or without treatment. Data are presented as mean ± standard deviation. IL-1β, interleukin-1 beta; MMP-13, matrix metalloproteinase; n = 5. *P < .05; **P < .01; and ***P < .001.

Besides the synovial tissue, cromolyn treatment significantly reduced (~5×) CD11b+ inflammatory cell migration to the labrum (Figure 8A). Labral IL-1β levels were also significantly reduced with cromolyn administration by ~2× and ~3× at weeks 1 and 3, respectively (Figure 8B). Moreover, cromolyn treatment reduced labral MMP-13 levels by ~1× and ~3× at weeks 1 and 3, respectively (Figure 8C).

### Effect of MC Stabilization on Labral Degeneration

**Figure 9.**
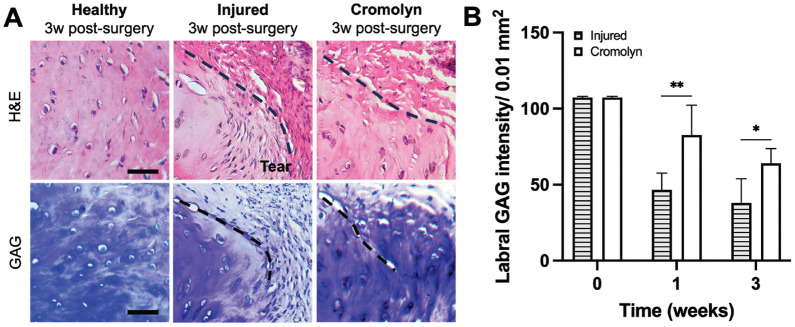
Effect of cromolyn on labral ECM degeneration after injury. (A) Representative images for toluidine blue (GAG) staining of healthy and injured labral tissue at 3 weeks. Scale bar = 50 µm. (B) Comparison of GAG intensity at 0, 1, and 3 weeks after labral injury. Data are presented as mean ± standard deviation. ECM, extracellular matrix; GAG, glycosaminoglycan; n = 5. *P < .05.

Finally, we analyzed the labral extracellular matrix (ECM) composition after injury with or without cromolyn treatment (Figure 9A). Injury alone led to extensive labral degeneration. At 3 weeks, MC stabilization reduced the extent of GAG loss in the labrum (Figure 9B). The cromolyn-treated labra exhibited a reduction of labral degradation as reflected in the loss of ECM (~30% more GAG preservation) compared with injured controls at 1 week (Figure 9B).

### Effect of MC Stabilization on Shoulder Functional Outcomes

**Figure 10.**
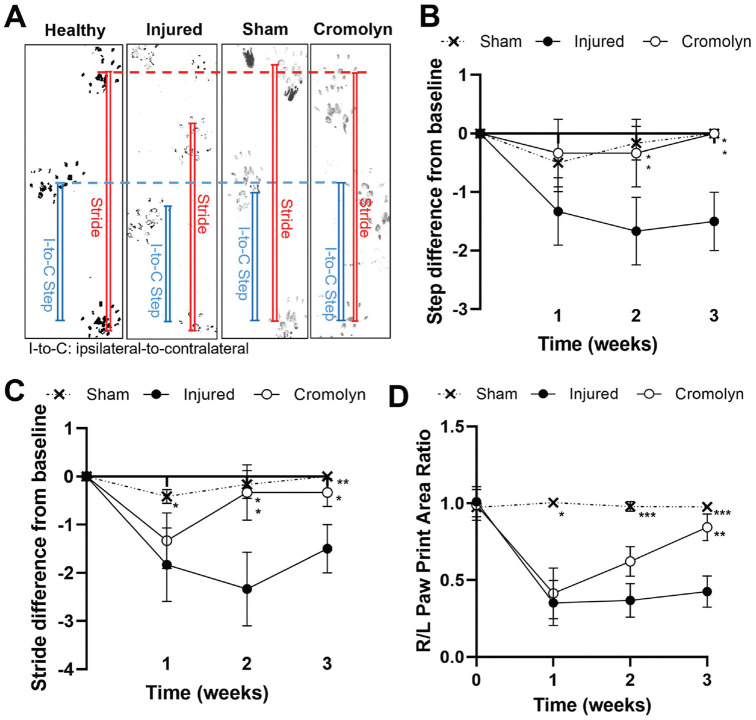
Longitudinal spatial gait analysis of rats with or without labral tears. (A) Visual demonstration of I-to-C step length and stride length of healthy, sham, injured, and cromolyn-treated rats after 3 weeks. (B) I-to-C step length difference from healthy baseline (0 weeks) up to 3 weeks after operation, (C) stride length difference, and (D) R/L paw print area ratio. Data are presented as mean ± standard deviation. I-to-C, ipsilateral to contralateral; R/L, right to left. *P < .05; **P < .01; ***P < .001. All statistical analyses are relative to the injured group of the same week.

The therapeutic effect of MC stabilization on the animals’ shoulder function after labral injury was assessed through ambulatory assessment (Figure 10A). The injured rats left untreated had a significantly greater decrease in their initial ipsilateral-to-contralateral step length (*P* = .0168), stride length (*P* = .0095), and ipsilateral to contralateral paw area ratio (*P* = .000311) at 3 weeks after surgery. Compared with healthy controls, the sham group showed insignificant step length differences and stride length differences after surgery (Figure 10, B and C), as well as similar ipsilateral to contralateral paw area ratios (*P* = .998) (Figure 10D**)**. The sham group's negligible difference from baseline and baseline's significant difference from the injured group demonstrates that the surgical procedure alone is not a strong confounder of shoulder functional outcomes. Most importantly, compared with injured rats at 3 weeks, the cromolyn-treated group had significant improvements in gait—*P* = .0167, *P* = .0989, *P* = .0014 for step, stride, and paw area ratio, respectively (Figure 10, B, C, and D)**—**supporting the significant role of MC activation on the deterioration of shoulder function after labral injury.

## Discussion

Synovial MCs are known drivers of inflammation and subsequent tissue destruction in other joint diseases. While histological analysis performed on human synovial tissue from several joints (shoulder, knee, hip, etc) demonstrated that diseased synovial membranes harbor significantly more activated MCs than healthy membranes,^22^ their specific contribution to shoulder labral inflammation and degeneration after injury has not been shown. Here, we showed that the labral tear significantly increased synovial MC activation, consistent with previous observations in humans showing high synovial MC activation in patients with type 2 SLAP lesions.^52^ The activation of MCs then elicited the recruitment and infiltration of inflammatory cells originating from synovial vasculature into the injured labrum. Because ~50% of cells in the inflamed synovia are CD11b+ cells at week 1, the influx of CD11b+ cells is likely to contribute to the increase of cellularity in inflamed synovia. During the migration of inflammatory cells and responses, abundant inflammatory products, such as IL-1β and MMP-13, were first found in the synovium and then in the injured labrum.

**Figure 11.**
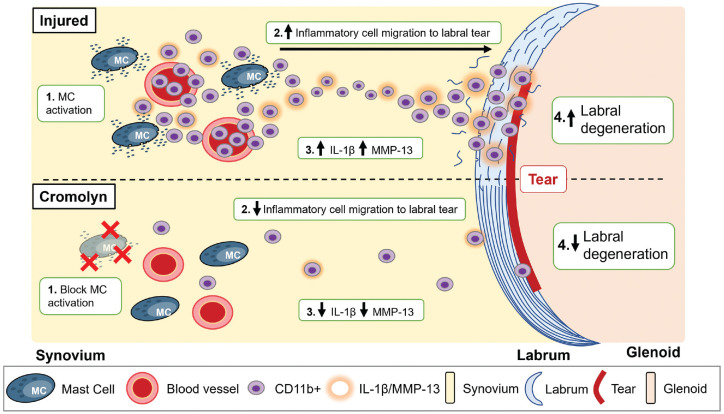
Summary of MC-mediated labral degeneration. The administration of cromolyn was able to block MC activation in the synovium and attenuate both synovial and labral inflammatory responses to reduce labral degeneration. IL-1β, interleukin-1 beta; MC, mast cell; MMP-13, matrix metalloproteinase.

These results align with previously published glenoid labral tear rat models, where TGF-β1, IL-1β, MMP-3, and MMP-13 expression increased in injured labra compared with healthy labra.^11,38^ Furthermore, IL-1β and MMP-13 exhibited localized distributions in the labrum, likely due to its proximity to the synovial membrane. IL-1β showed strong staining at the labral edges at 1 week and later showed more diffuse expression in the labral matrix (3 weeks). On the other hand, MMP-13 had a similar trend and was distributed throughout the labral matrix at 1 week, suggesting that chondrocyte activation contributed to labral MMP-13 expression. Elevated inflammatory products, such as TNF-α and IL-1β, have been shown to increase the production of ECM-degrading enzymes by labral cells, such as MMP-1, MMP-2, MMP-9, and ADAMTS-4.^14^ While the overall processes of labral injury–induced labral degeneration might be multifactorial (ie, excessive instability and mechanical breakdown of unhealed labrum), our results highlight 1 of those processes in which labral tear induces MC activation, followed by inflammatory cell recruitment, inflammatory products release, ECM degrading enzyme release, and labral tissue degradation (Figure 11).

To investigate the role of MC activation in labral degeneration after labral tear, animals were treated with cromolyn sodium, an MC membrane stabilizer, to prevent the release of granular products of MCs such as histamine. Cromolyn is a Food and Drug Administration–approved medication for treating allergic and inflammatory conditions such as asthma and MC disorders.^37^ With cromolyn treatment, synovial MC activation was reduced, leukocyte infiltration in injured labra was reduced, and both synovial and labral expression of IL-1β and MMP-13 were reduced after injury (Figure 11). Consequently, there was a reduction in labral GAG loss and preservation of labral structural integrity. Equally important, reducing inflammatory responses with cromolyn treatment allowed rats to recover their preoperative gait patterns. These results suggest that MC stabilizers may be a promising treatment adjuvant for limiting acute inflammation and symptomatic management after labral tears. Notably, cromolyn given at the current dose did not completely inhibit MC activation. Further studies would be needed to determine the optimal dosage for full MC stabilization or whether other pathways are involved in MC activation and subsequent labral degeneration. Other drugs, such as tranilast and ketotifen, have been used as MC stabilizers by suppressing histamine release.^1^ Our results are consistent with previously published studies investigating the effect of MC stabilization and tissue destruction of various joints. Cyclic adenosine monophosphate-inducing compound salbutamol and cromolyn efficiently protected against avβ3 integrin activation, angiogenesis, pannus formation, and ankle joint destruction in a mouse model of glucose-6-phosphate isomerase–induced arthritis.^28^ Additionally, treatment with MC stabilizer sodium nedocromil in a mouse collagen-induced arthritis model was associated with significant reductions in neutrophil infiltration in digit and knee samples.^42^ A recent large observational study in humans showed patients treated with H_1_-antihistamines, which can stabilize MCs and have anti-inflammatory effects, had a lower prevalence of radiographic knee osteoarthritis.^49^

This study has several limitations. First, the current animal model might not perfectly replicate the clinical conditions of labral tears in humans. Rats are frequently chosen as animal models for shoulder pathology because of their anatomic resemblance to humans^21^; nonetheless, it is essential to conduct further investigations to validate our results in humans. Consequently, evaluating shoulder function plays a pivotal role in assessing the potential translation of our approach. Despite the difference in gait between 4-limbed animals and humans, similar factors influence the response of shoulders to injuries.^34^ Second, while the torn labrum was created and then repaired using a bone tunnel suture technique to simulate current clinical practice in humans, the suture anchor hole in the surgical procedure was placed close to the center/bare spot of the glenoid, which is not typically done in the clinic. We assessed the influence of the suture anchor (drill hole and suture) on inflammatory responses and carried out studies without a suture anchor.^11^ Our results showed no significant differences in inflammatory responses between treatment groups with and without a suture anchor at 3 weeks (Appendix Figure A2, available online). These results suggest a suture anchor has minimal effect on labral tear–induced inflammatory responses. Moreover, the surgical procedures created large surgical trauma because of the lack of minimally invasive instruments for rodents. MC activation may also be triggered by both surgical trauma and labral injury. However, our results show that only labral injury, but not surgical trauma, can trigger lasting (or 3 weeks) inflammatory responses. Third, cromolyn has been used in humans for many years and has a well-established safety profile.^37^ However, whether MC stabilization or reducing synovial inflammation influences stem cell responses and labral healing has yet to be determined. While we demonstrated inflammatory responses prompt labral degeneration in the short term, further studies are necessary to determine the relationship between inflammation and labral healing in the long term. Finally, cromolyn was administered systemically. Potential off-target effects were not evaluated. The local administration of cromolyn has not been studied; however, it may be of future interest.

## Conclusion

Our results demonstrated that MC activation plays a pivotal role in eliciting synovial and labral inflammatory responses, leading to significant labral ECM degradation, labral degeneration, and shoulder function deterioration after injury. The administration of cromolyn can effectively reduce synovial/labral inflammation, labral degeneration, and shoulder functional recovery after injury. These findings support the idea that an MC stabilizer may be used as a complementary therapeutic option in the treatment and repair of labral tears.

## Supplemental Material

sj-pdf-1-ajs-10.1177_03635465241278671 – Supplemental material for Mast Cells Mediate Acute Inflammatory Responses After Glenoid Labral Tears and Can Be Inhibited With Cromolyn in a Rat Model Supplemental material, sj-pdf-1-ajs-10.1177_03635465241278671 for Mast Cells Mediate Acute Inflammatory Responses After Glenoid Labral Tears and Can Be Inhibited With Cromolyn in a Rat Model by Cynthia M. Co, Bhavya Vaish, Le Q. Hoang, Tam Nguyen, Joseph Borrelli, Jr, Peter J. Millett and Liping Tang in The American Journal of Sports Medicine
