# Supplementary material for: Mast Cells Mediate Acute Inflammatory Responses After Glenoid Labral Tears and Can Be Inhibited With Cromolyn in a Rat Model
Source: Am J Sports Med. 2024 Oct 6;52(13):3357–69. doi: 10.1177/03635465241278671 (PMC11542330; doi:10.1177/03635465241278671)
Supplement: sj-pdf-1-ajs-10.1177_03635465241278671 – Supplemental material for Mast Cells Mediate Acute Inflammatory Responses After Glenoid Labral Tears and Can Be Inhibited With Cromolyn in a Rat Model [file sj-pdf-1-ajs-10.1177_03635465241278671.pdf]

Mast Cells Mediate Acute Inflammatory Responses After Glenoid Labral Tears and Can be Inhibited with Cromolyn in a Rat Model

APPENDIX

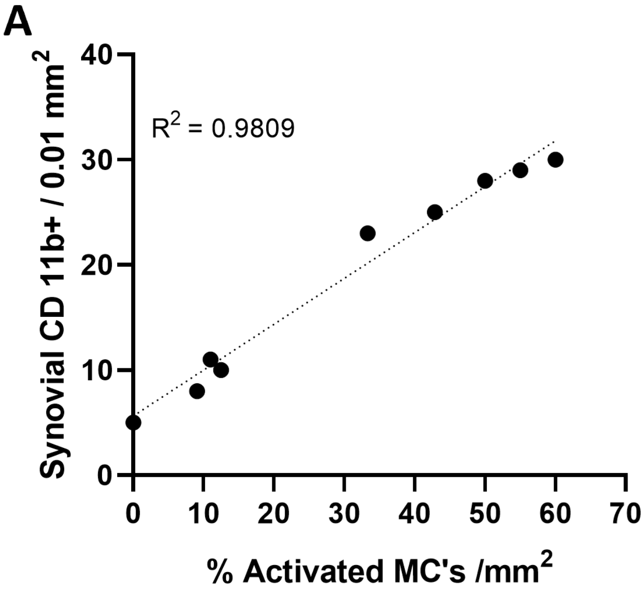

Figure A1. Linear regression analysis between activated MC and synovial CD 11b+ 4 inflammatory cells in the rats treated with compound 48/80.

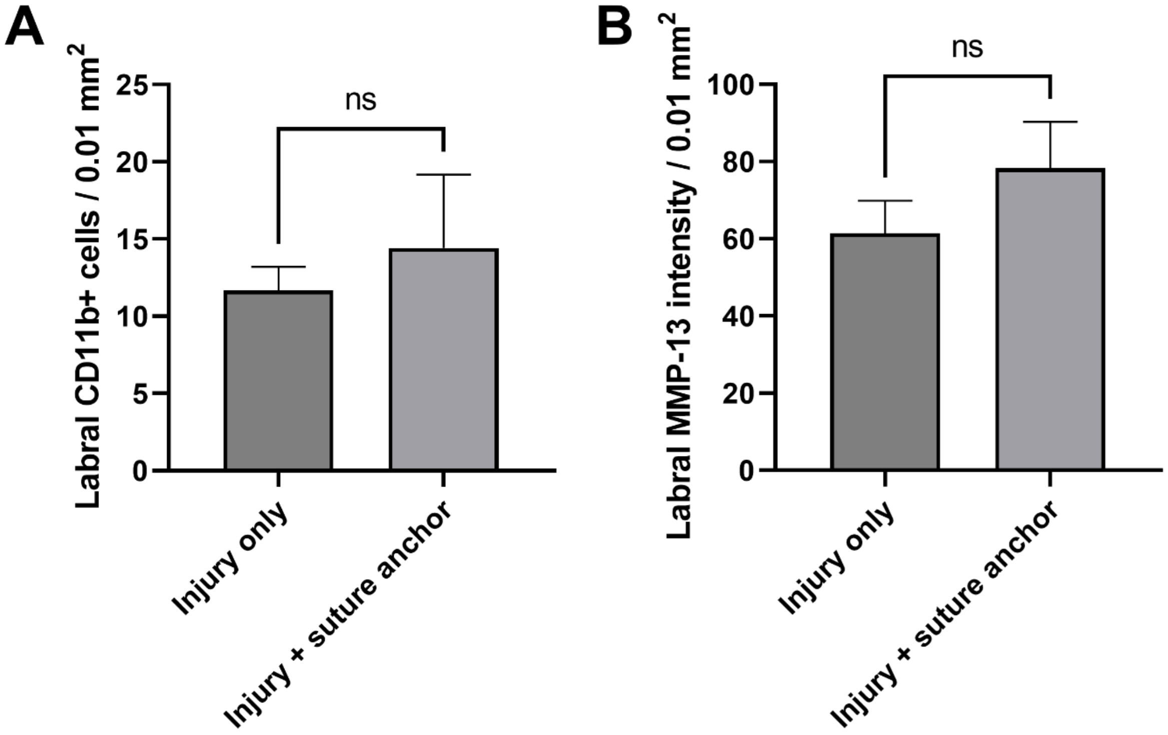

Figure A2. Inflammatory responses following labral injury with or without suture anchor 8 at 3 weeks. (A) Number of CD11b+ cells, and (B) MMP-13 intensity in the injured labrum. Data 9 presented as mean ± SD, n=3. ns = not significant
